# Atomic force microscopy reveals involvement of the cell envelope in biomechanical properties of sickle erythrocytes

**DOI:** 10.1186/s12915-023-01523-3

**Published:** 2023-02-13

**Authors:** Kun Wang, Zhiqiang Li, Ogechukwu Egini, Raj Wadgaonkar, Xian-Cheng Jiang, Yong Chen

**Affiliations:** 1 Jiangxi Key Laboratory for Microscale Interdisciplinary Study, Institute for Advanced Study, Nanchang University, Nanchang, Jiangxi 330031 People’s Republic of China; 2 Department of Cell Biology, SUNY Health Sciences University, State University of New York, Brooklyn, NY 11203 USA; 3 Division of Hematology and Oncology, Department of Medicine, SUNY Health Sciences University, State University of New York, Brooklyn, NY 11203 USA; 4 VA Medical Center, Brooklyn, NY11208 USA

**Keywords:** Sickle cell anemia (SCA), Sickle erythrocytes, Atomic force microscopy (AFM), Membrane ghosts, Methyl-β-cyclodextrin (MβCD), Stiffness, Membrane-associated hemoglobin

## Abstract

**Background:**

Intracellular hemoglobin polymerization has been supposed to be the major determinant for the elevated rigidity/stiffness of sickle erythrocytes from sickle cell anemia (SCA) patients. However, the contribution of the cell envelope remains unclear.

**Results:**

In this study, using atomic force microscopy (AFM), we compared the normal and sickled erythrocyte surfaces for stiffness and topography. AFM detected that sickle cells had a rougher surface and were stiffer than normal erythrocytes and that sickle cell ghosts had a rougher surface (for both outer and inner surfaces) and were thicker than normal ghosts, the latter implying a higher membrane-associated hemoglobin content/layer in the sickle cell envelope. Compared to healthy subjects, the SCA patients had lower plasma lipoprotein levels. AFM further revealed that a mild concentration of methyl-β-cyclodextrin (MβCD, a putative cholesterol-depleting reagent) could induce an increase in roughness of erythrocytes/ghosts and a decrease in thickness of ghosts for both normal and sickle cells, implying that MβCD can alter the cell envelope from outside (cholesterol in the plasma membrane) to inside (membrane-associated hemoglobin). More importantly, MβCD also caused a more significant decrease in stiffness of sickle cells than that of normal erythrocytes.

**Conclusions:**

The data reveal that besides the cytosolic hemoglobin fibers, the cell envelope containing the membrane-associated hemoglobin also is involved in the biomechanical properties (e.g., stiffness and shape maintenance) of sickle erythrocytes.

**Supplementary Information:**

The online version contains supplementary material available at 10.1186/s12915-023-01523-3.

## Background

Sickle cell disease is the most common severe monogenic disease with a single nucleotide mutation (GAG codon changing to GTG) at the β-globin gene in chromosome 11 [1]. Every year, ~300,000 infants are born with sickle cell anemia globally but particularly in tropical regions including sub-Saharan Africa (~80%), tribal regions of India, and the Middle East [2]. At present, the average life expectancy of sickle cell anemia patients is up to 60 years in developed countries [2]. In sickle cells, hemoglobin A (HbA) comprised of two α chains and two β chains is replaced by hemoglobin S (HbS) in which the glutamic acid (E/Glu) at position 6 of the β chain is substituted by valine (V/Val) due to the single nucleotide mutation [1]. Under a condition of low O_2_ concentration, the hydrophobic side chain of the valine residue is able to cause the aggregation/polymerization of deoxygenated HbS (deoxy-HbS) molecules and the formation of fibrous precipitates, resulting in a sickle shape of red blood cells (or erythrocytes) which only last 10–20 days in blood circulation [1].

The loss of elasticity (or the increase of rigidity/stiffness) of sickle cells is central to the pathophysiology of the disease since the rigid erythrocytes are unable to pass through narrow capillaries due to the lack of deformability, resulting in vessel occlusion, ischemia, and hemolysis, among others (although surface area-to-volume ratio was found to be a major determinant of erythrocyte traversal [3]). Therefore, it is very important to detect the biomechanical properties of sickle cells and reveal the determinants of sickle erythrocyte rigidity. Generally, increased intracellular HbS concentration (or intracellular polymerization of HbS) inside sickle cells is regarded as the major contributor for the elevated rigidity of sickle cells [4–6]. However, it remains unclear whether the sickle cell envelope composed of the plasma membrane, the membrane skeleton, and the membrane-associated hemoglobin is responsible for the elevated rigidity of sickle cells.

Many techniques are available for measuring the elasticity or rigidity/stiffness of individual cells, including micropipette aspiration, atomic force microscopy (AFM), optical tweezer, and others, among which AFM has long been widely applied in the imaging and biomechanical (e.g., rigidity/stiffness [7–10], adhesion [11], and protein-erythrocyte interaction [12–15]) detection of sickle erythrocytes due to the high-resolution (nanoscale) imaging and high-sensitivity force measurement functions of AFM under physiological conditions. In this study, AFM was recruited to investigate the involvement of sickle cell envelope in the contribution to the elevated rigidity of sickle cells by imaging the entire morphology and surface ultrastructure/roughness of erythrocytes and the roughness and thickness of isolated membrane ghosts and by measuring the stiffness (Young’s modulus) of entire erythrocytes. Considering cholesterol is the well-known major molecule responsible for regulating the rigidity of the plasma membrane of animal cells, methyl-β-cyclodextrin (MβCD, a putative cholesterol-depleting reagent) also was utilized to decrease the cholesterol level in the plasma membrane of normal and sickle erythrocytes.

## Results

### Lower lipid levels in sera/lipoproteins of sickle cell anemia (SCA) patients compared with the healthy controls

**Fig. 1:**
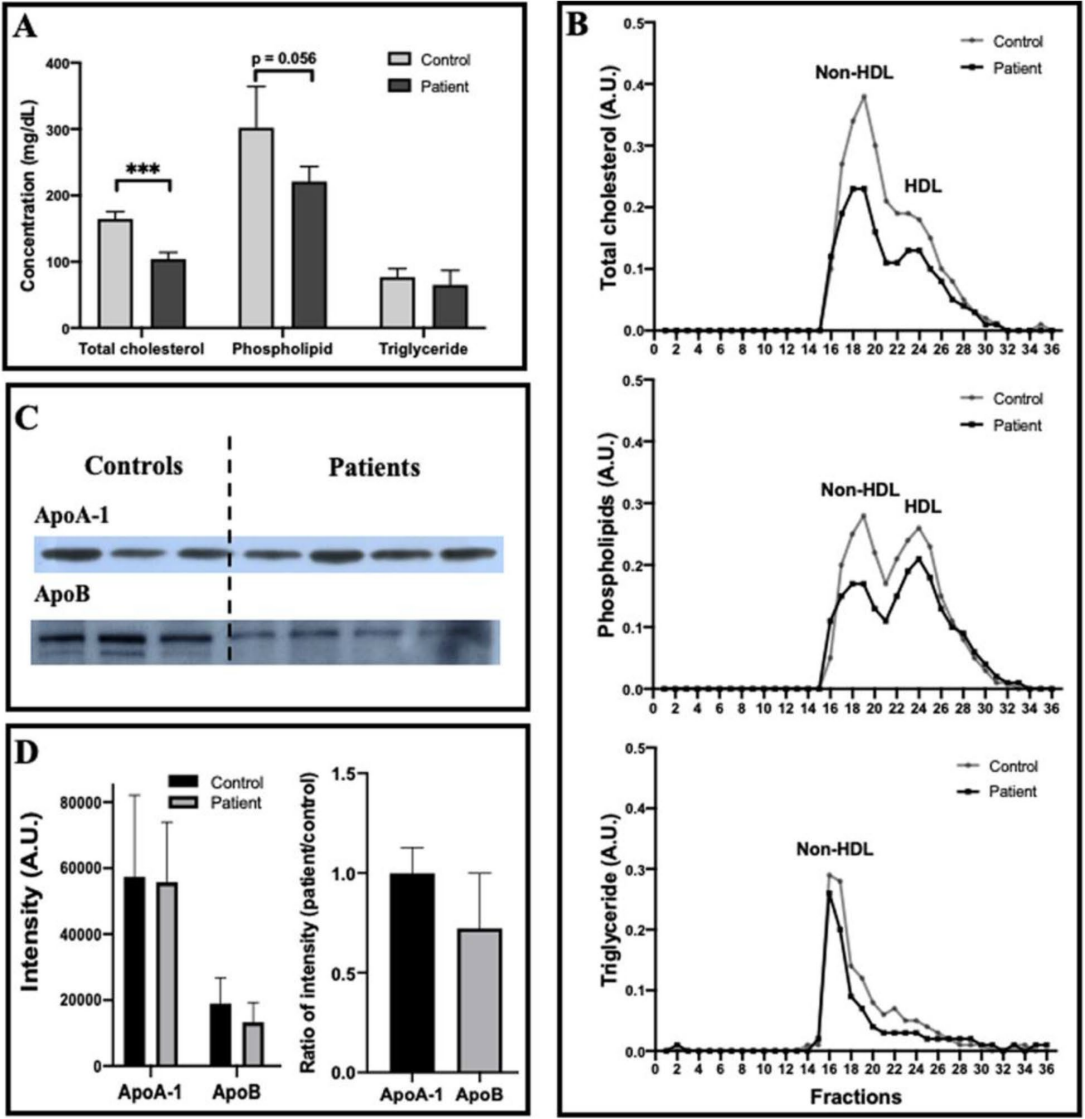
Lipid profiles, the masses of apoA-I and apoB in serum of sickle cell anemia (SCA) patients compared with healthy human subjects (the controls). **A** Lipid profiles of sera including total cholesterol, phospholipid, and triglyceride. **B** Lipid profiles of lipoproteins (non-HDL and HDL) isolated from sera via FPLC. **C** Representative western blots of ApoA-I and ApoB (The molecular weights of human ApoA-I and ApoB-100 are ~28.3 kDa and ~510 kDa, respectively). **D** Quantitative analyses of the western blot data. In **A** and **D**, *n* = 3 for healthy subjects and *n* = 4 for SCA patients. Statistical significance is indicated as follows: ***P < 0.001

Prior to the detection of erythrocytes, lipid profiling of sera from 3 healthy controls and 4 SCA patients was performed. Compared with healthy subjects, SCA patients had lower concentrations of three major lipids including total cholesterol (104.2 ± 9.7 mg/dL for SCA vs. 164.6 ± 10.8 mg/dL for controls), phospholipids (221.0 ± 22.5 mg/dL for SCA vs. 302.1 ± 62.0 mg/dL for controls), and triglyceride (65.0 ± 22.0 mg/dL for SCA vs. 76.7 ± 12.9 mg/dL for controls) although the latter two did not reach a statistical significance (Fig. 1A).

Lipids in human blood are delivered mainly via plasma lipoproteins including ApoA-I-containing lipoproteins (e.g., high-density lipoprotein or HDL) and ApoB-containing lipoproteins (e.g., non-HDL including very low-density lipoprotein or VLDL, low-density lipoprotein or LDL, etc.). To detect the lipid profiles in plasma lipoproteins, aliquots of sera from 3 healthy controls or 4 SCA patients were mixed, and approximately 36 fractions including HDL and non-HDL lipoproteins were separated by a fast protein liquid chromatography (FPLC) which were subjected to lipid profiling via fluorescence spectrometry. The data show that the three lipids (total cholesterol, phospholipids, and triglyceride) in both HDL and particularly non-HDL lipoproteins of SCA patients decreased in comparison with those in the healthy controls (Fig. 1B).

Interestingly, The distribution of phospholipid contents in HDL and non-HDL lipoproteins changed. In the healthy controls, the phospholipid content in HDL is slightly lower than that in non-HDL (the gray curve in the middle panel of Fig. 1B); in the SCA patients, however, the phospholipid level in HDL is slightly higher than that in non-HDL (the black curve in the middle panel of Fig. 1B).

### Lower ApoB level whereas similar ApoA-I level in sera of SCA patients compared with the controls

Then, the masses of the major apolipoproteins constructing HDL and non-HDL lipoproteins were evaluated via western blotting. Both the representative western blot bands (Fig. 1C) and the quantitative analyses (Fig. 1D) of ApoA-I and ApoB masses in sera of 3 healthy controls and 4 SCA patients show that ApoB level in SCA patients was much lower, although not statistically significant due to small sample size (3–4 samples), than that in healthy controls (*p* = 0.0731 in the left panel of Fig. 1D; a patient/control ratio of 0.723 ± 0.276 in the right panel of Fig. 1D) whereas ApoA-I level was similar (*p* = 0.8586 in left panel of Fig. 1D; a patient/control ratio of 0.997 ± 0.129 in the right panel of Fig. 1D).

### Sickle erythrocytes have a rougher surface than normal erythrocytes detected by AFM

**Fig. 2:**
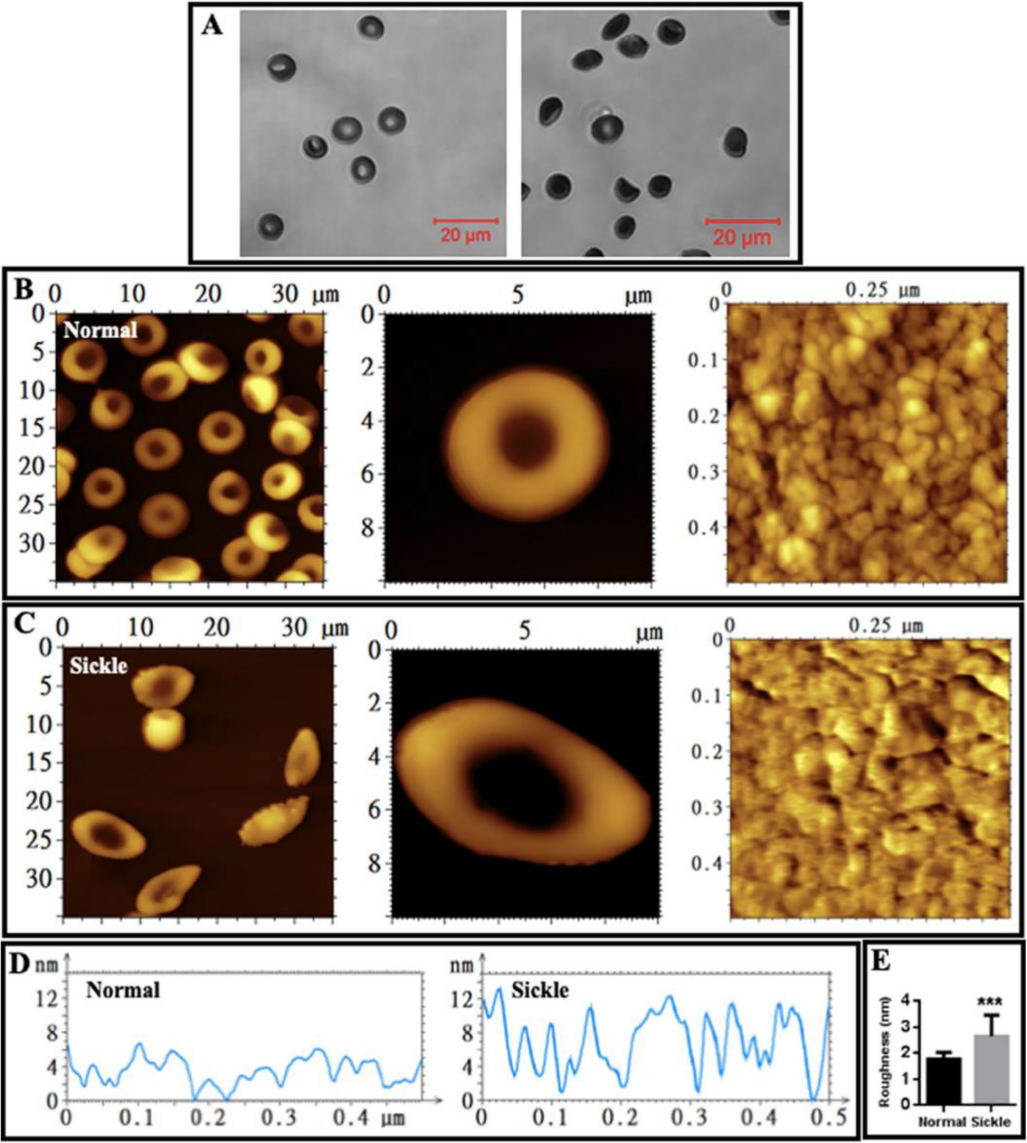
AFM topographical images of erythrocytes fixed by glutaraldehyde. **A** Morphology of individual normal erythrocytes (left) and sickle erythrocytes (right) observed by optical microscopy. **B** AFM images of normal erythrocytes. Left: multiple erythrocytes; middle: single representative erythrocyte; right: local structures on a single normal erythrocyte. **C** AFM images of sickle erythrocytes from sickle cell anemia patients. Left: multiple erythrocytes; middle: single representative erythrocyte; right: local structures on a single sickle erythrocyte. **D** High profiles of the cross sections across the local structures on a single normal (left) or sickle (right) erythrocyte. **E** Roughness quantification of the local structures on single normal or sickle erythrocyte detected by AFM (*n* = 25; ***P < 0.001)

Next, red blood cells (RBCs or erythrocytes) isolated from healthy subjects and SCA patients were observed with confocal microscopy prior to the observation by atomic force microscopy (AFM). The typical biconcave erythrocytes and sickle cells were observed in healthy controls and SCA patients, respectively (Fig. 2A). Subsequently, AFM studies of erythrocytes were performed. The typical biconcave erythrocytes and sickle erythrocytes from healthy subjects and SCA patients, respectively, were also observed by AFM (left and middle panels of Fig. 2B, C). The topographical AFM images of local surfaces of individual cells (right panels of Fig. 2B, C) and the height profiles of cross sections across the local surfaces (Fig. 2D) display a relatively rougher surface of sickle erythrocytes compared with normal erythrocytes. The quantitative analysis (Fig. 2E) further confirms the statistically significant increase in surface roughness of sickle erythrocytes (the average roughness is 1.8 ± 0.3 nm and 2.6 ± 0.8 nm for normal and sickle erythrocytes, respectively).

### Sickle cell membrane ghosts contain a higher hemoglobin level than normal ghosts detected by SDS-PAGE

**Fig. 3:**
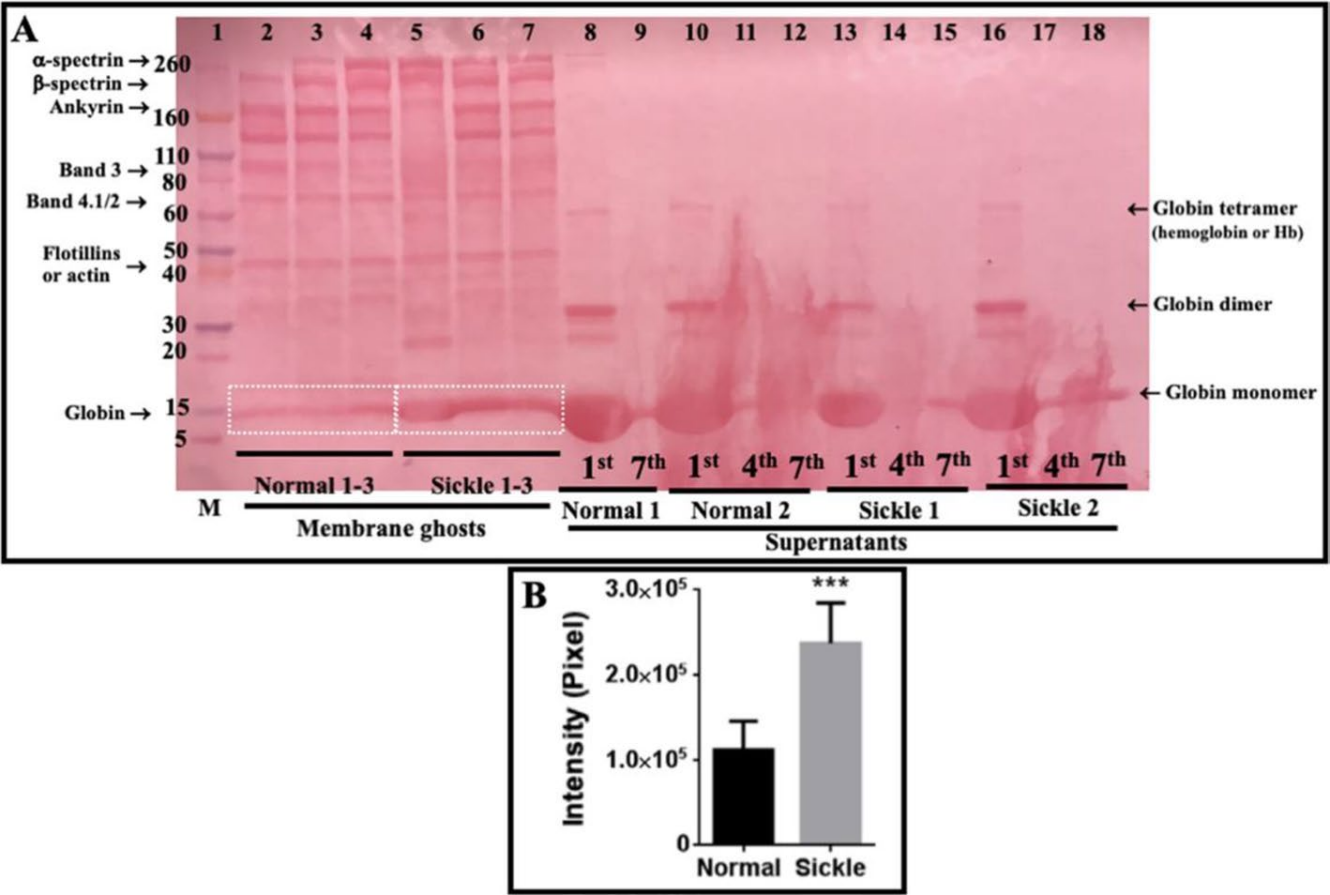
Major proteins in isolated membrane ghosts and in supernatants after centrifugation of membrane ghosts of normal and sickle erythrocytes. The samples were separated by SDS-PAGE, transferred to nitrocellulose membrane, and stained with ponceau S (**A**). Lane 1: protein marker; lanes 2–4: isolated membrane ghosts of the normal erythrocytes from three healthy subjects; lanes 5-7: isolated membrane ghosts of the sickle erythrocytes from three sickle cell anemia patients; lanes 8–12: the supernatants after 1^st^, 4^th^, and 7^th^ centrifugation of membrane ghosts of normal erythrocytes from two healthy subjects; lanes 13–18: the supernatants after 1^st^, 4^th^, and 7^th^ centrifugation of membrane ghosts of sickle erythrocytes from two patients. **B** Quantitative analysis of the hemoglobin bands indicated by the white boxes in **A** (***P < 0.001)

The entire cell envelope (generally called “membrane ghosts” or “ghosts”) of erythrocytes can be easily prepared/isolated from erythrocytes via hypotonic treatment and repeated centrifugation for specific studies on the erythrocyte membrane. Prior to AFM detection, the major proteins in isolated membrane ghosts and in the supernatants after centrifugation of membrane ghosts were separated by SDS-PAGE, transferred to nitrocellulose membrane, and visualized after ponceau S staining (Fig. 3A). The supernatants after hypotonic treatment and the first-round centrifugation contained most globin proteins (including monomer, dimer, and tetramer/hemoglobin due to SDS treatment for gel electrophoresis), after the 7^th^ round of centrifugation a few globin monomers still could be detected in the supernatants (lanes 8–18 in Fig. 3A). There were no obvious differences in protein type/content of supernatants between normal and sickle samples. The membrane ghosts (hypotonic treatment and 7 rounds of centrifugation) from all samples contained multiple proteins/bands (some specific proteins including α-/β-spectrin, ankyrin, band 3, globin, and others are indicated in the image based on their molecular weights and the locations of their bands corresponding to the marker bands), among which the ghosts from sickle erythrocytes exhibited a higher level of hemoglobin in comparison with the ghosts from normal erythrocytes (lanes 2–7 in Fig. 3A) which was further confirmed by the quantitative analysis (Fig. 3B).

### Sickle erythrocyte membrane ghosts are thicker and rougher than normal ghosts detected by AFM

**Fig. 4:**
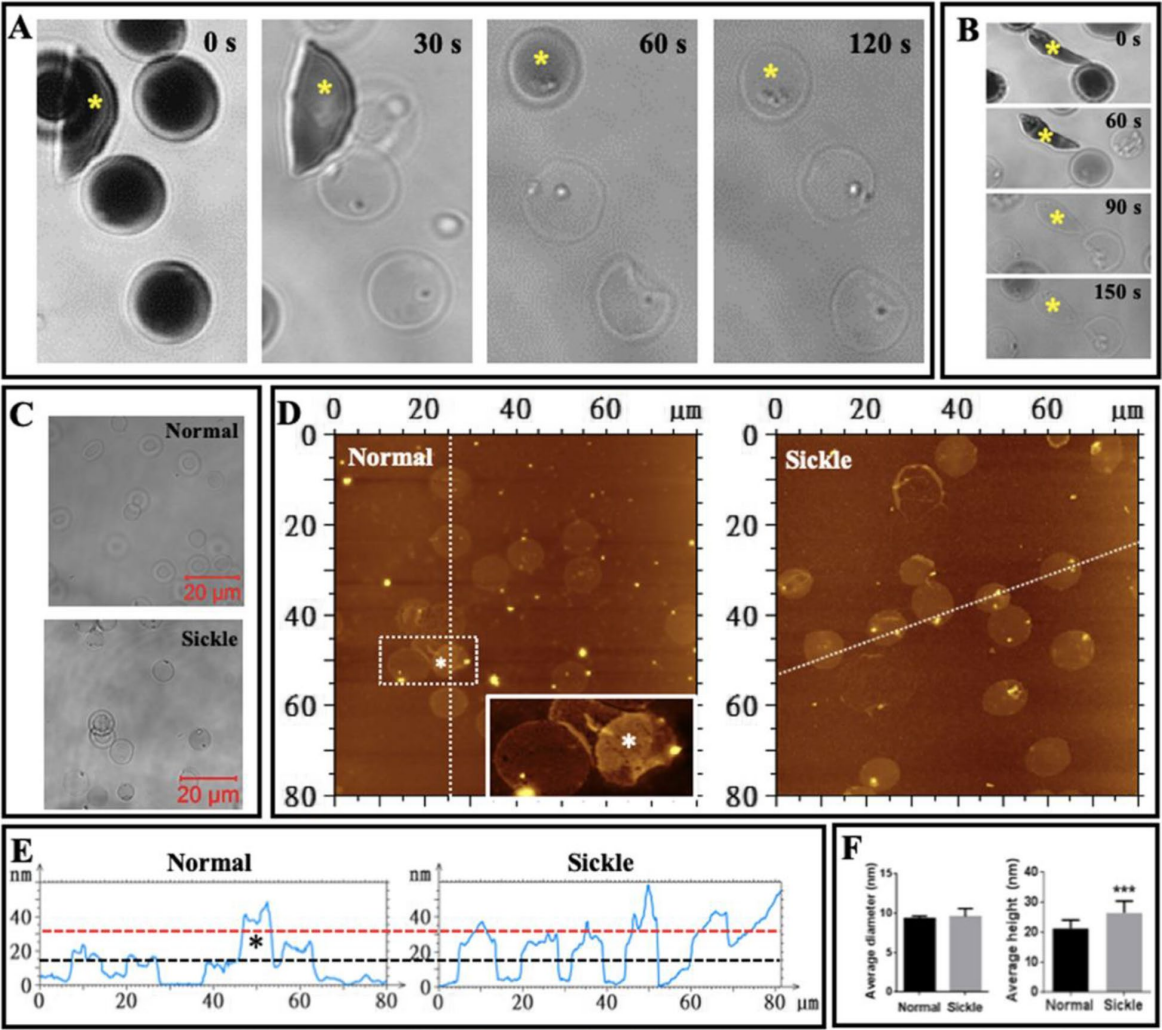
Optical and AFM imaging of entire erythrocyte membrane ghosts. **A**, **B** Time-lapse optical observation of the transformation from whole cells (dark) to membrane ghosts (ghosts; transparent) in hypotonic solution. The yellow asterisks indicate the sickle-shaped cells/ghosts. **C** Optical observation of membrane ghosts from normal (top) and sickle (bottom) erythrocytes. **D** AFM topographical images of glutaraldehyde-fixed ghosts from normal erythrocytes (left; the inset is enlarged from the white square showing the overlapping of two ghosts indicated by the white asterisks) and sickle erythrocytes (right). **E** Height profiles of the cross sections of individual ghosts across the dashed lines in **C** (the black asterisk in **D** corresponds to the white asterisks in **C**). **F** Quantitative analysis of average diameter (left) and average height (right) of ghosts (*n* = ~50; ***P < 0.001)

Real-time optical observation shows that erythrocytes became transparent after the removal of intracellular hemoglobin (Fig. 4A–C). It was observed that the transformation of most sickle erythrocytes into membrane ghosts lagged behind in comparison with that of normal erythrocytes (Fig. 4A) and some sickle cells temporarily maintained a sickle shape after the removal of intracellular hemoglobin (Fig. 4B). Eventually, however, almost all sickle erythrocytes became membrane ghosts with a wafer-shaped (or circular pie-shaped) morphology similar to those of normal erythrocytes as observed by optical microscope (Fig. 4C) and AFM (Fig. 4D).

The height profiles of the cross sections across multiple entire ghosts in AFM topographical images show that individual ghosts from sickle erythrocytes were higher than those from normal erythrocytes although they have a similar diameter (Fig. 4E; the black and red dashed lines indicate the approximate heights of normal and sickle cell membrane ghosts, respectively). Quantitative analyses find that the average ghost diameters from normal and sickle erythrocytes are 9.09 ± 0.57 μm and 9.54 ± 1.04 μm, respectively and that the average ghost heights from normal and sickle erythrocytes are 21.3 ± 2.8 nm and 26.4 ± 4.0 nm, respectively (Fig. 4F), which actually contain two layers of membrane ghost.

**Fig. 5:**
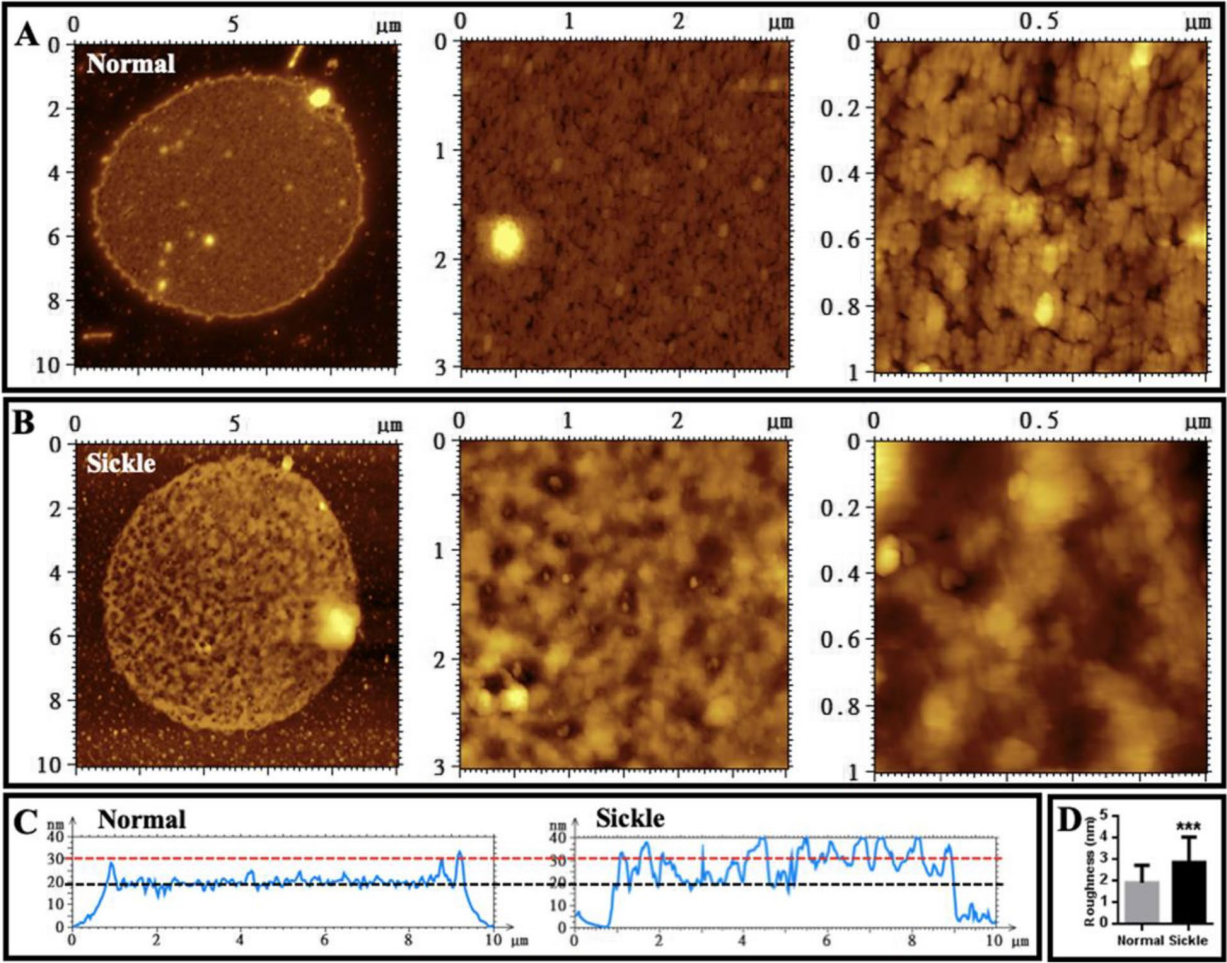
AFM topographical images of local structures of individual erythrocyte membrane ghosts fixed by glutaraldehyde. **A** A representative membrane ghost of normal erythrocytes. **B** A representative membrane ghost of erythrocytes from sickle cell anemia patients. From left to right panels: Entire membrane ghosts, local structures (3 μm × 3 μm), and smaller local structures (1 μm × 1 μm), respectively. **C** Height profiles of the cross sections of individual normal (left) and sickle (right) membrane ghosts. **D** Quantitative analysis of average roughness of the local surfaces (*n* = 30; ***P < 0.001)

When single membrane ghosts were observed by AFM and local structures were highlighted, it was found that the surface of membrane ghosts from normal erythrocytes (Fig. 5A) seems much smoother than that from sickle erythrocytes (Fig. 5B). It is worth noticing that many small bumps appear on the surface of membrane ghosts from sickle erythrocytes (Fig. 5B). The height profiles of the cross sections across single entire ghosts confirm that compared with normal erythrocytes sickle cells have a membrane ghost with similar diameter, higher thickness (Fig. 5C; the black and red dashed lines indicate the approximate heights of normal and sickle cell ghosts, respectively), and rougher surface. The quantitative analysis (Fig. 5D) reveals the statistically significant increase in average surface roughness of membrane ghosts from sickle erythrocytes (1.9 ± 0.8 nm and 2.9 ± 1.2 nm for the membrane ghosts from normal and sickle erythrocytes, respectively). The average surface roughness values of membrane ghosts are similar to those of entire erythrocytes (Fig. 2E).

**Fig. 6:**
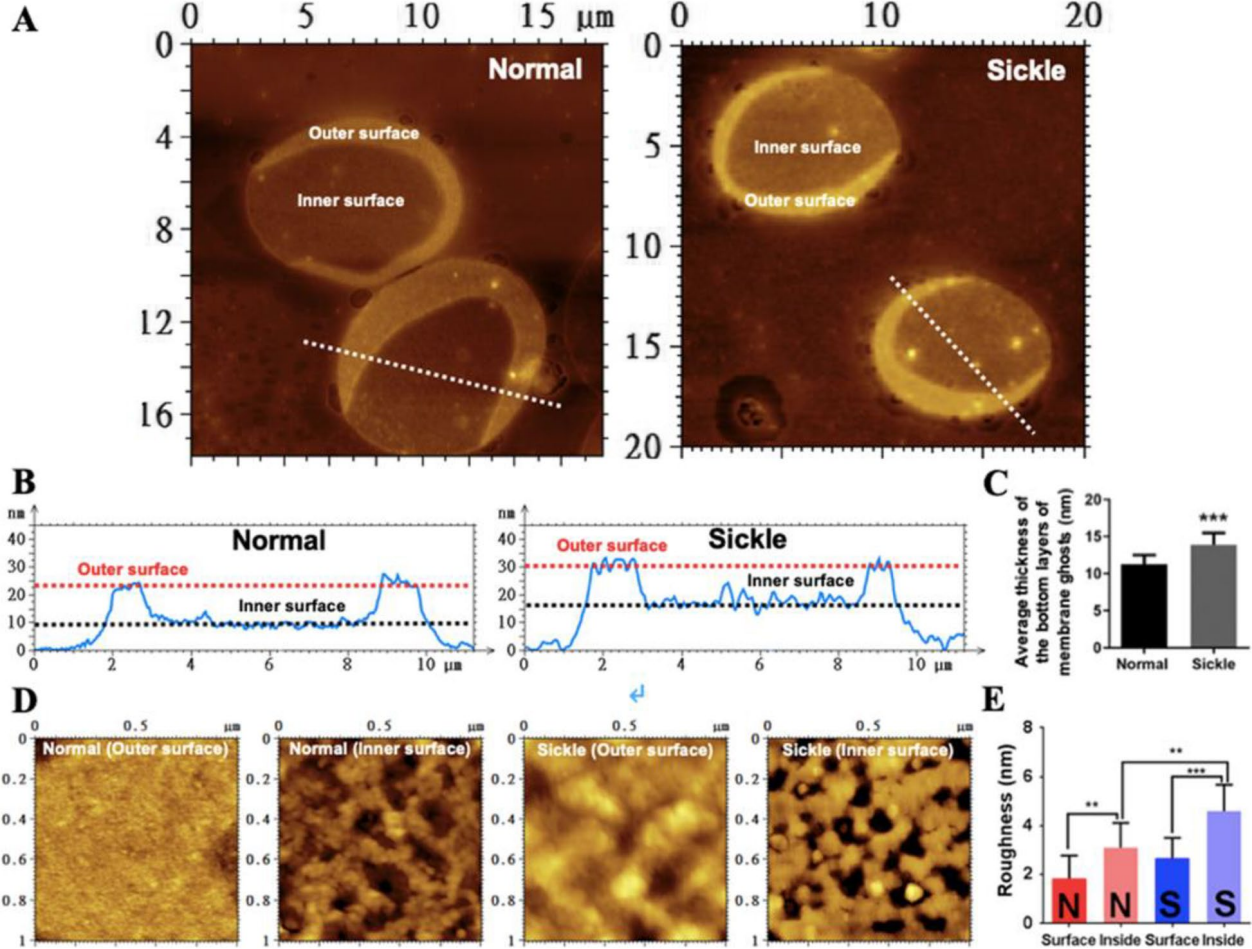
AFM topographical images of outer and inner/cytoplasmic surfaces of individual erythrocyte membrane ghosts. **A** Representative membrane ghosts of normal (left) and sickle (right) erythrocytes. **B** Height profiles of the cross sections of individual normal (left) and sickle (right) ghosts showing the heights of the inner and outer surfaces which are indicated by the black and red dashed lines, respectively. **C** Quantification of the average thickness of the bottom layers of membrane ghosts (*n* = 15; ***P < 0.001). **D** Representative topographical images showing the local ultrastructures of the outer and inner surfaces of normal and sickle erythrocytes. The meshwork-like structures with a mesh size of less than 200 nm are evident in the inner surfaces of both normal and sickle cell ghosts. **E** Quantitative analysis of average roughness of the local surfaces (*n* = 15; **P < 0.01, ***P < 0.001). The marks N and S represent the normal and sickle cell samples, respectively

To visualize the inner surface of membrane ghosts, most of the upper layer of membrane ghosts was peeled by shear force to expose the inner/cytoplasmic surface of the bottom layer of membrane ghosts remaining a small part of the upper layer of membrane ghosts with its outer surface detectable by AFM (Fig. 6A). The height profiles of cross sections (Fig. 6B) show that the heights of the membrane ghost in one layer from normal and sickle cells are approximately 10 nm and 15 nm, respectively. the quantitative analysis (Fig. 6C) shows the average thickness of the bottom layers of membrane ghosts (11.3 ± 1.2 nm for normal erythrocyte membrane vs. 13.9 ± 1.6 nm for sickle cell membrane) which are around half of the heights of the intact membrane ghost in two layers (Fig. 4F). The data confirms that the ghosts in one layer from sickle erythrocytes are thicker than those from normal erythrocytes. Figure 6D displays the representative AFM topographical images of the outer and inner surfaces of membrane ghosts from normal and sickle erythrocytes. It is worth noticing that the inner/cytoplasmic surfaces of both normal and sickle cell ghosts display a meshwork-like structure which probably is based on the spectrin network beneath the plasma membrane. Both the topographical images and the quantitative analysis of average surface roughness (Fig. 6E) show that the inner surface is rougher than the outer surface for both normal and sickle ghosts and that sickle ghosts are rougher than normal ghosts for both outer and inner surfaces.

### Cholesterol depletion by MβCD induces surface roughening at relatively low concentration and surface damage or even cell degradation at high concentrations for both normal and sickle erythrocytes

**Fig. 7:**
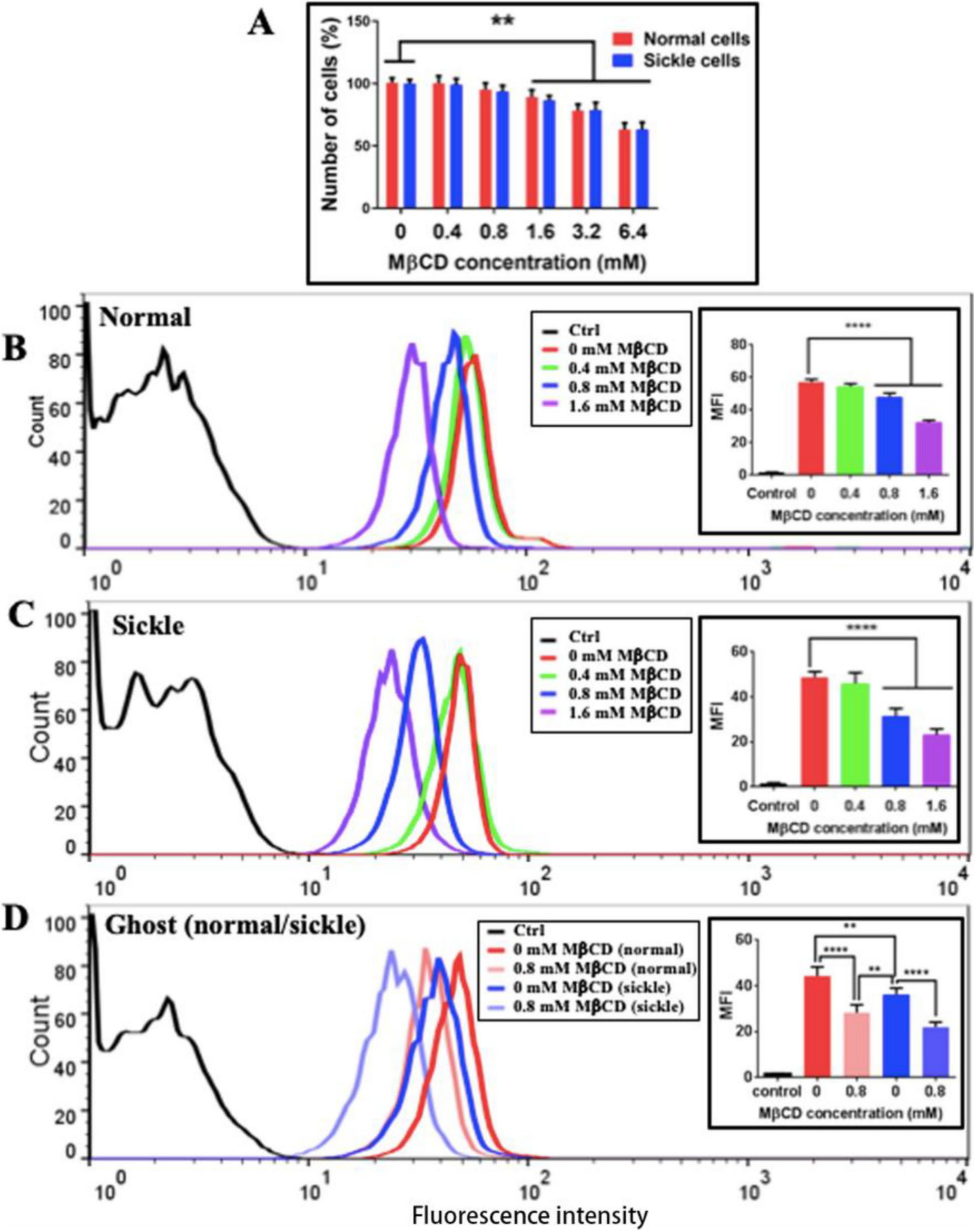
Effects of MβCD at different concentrations on cell viability and cholesterol depletion. **A** Effect of MβCD on the number of erythrocytes (*n* = 6; **P < 0.01). **B**–**D** The representative flow cytometric data show the fluorescence intensity of filipin-stained normal erythrocytes (**B**), sickle erythrocytes (**C**), and their membrane ghosts (**D**), respectively, treated with MβCD at indicated concentrations for 1h at 37 °C. Insets show the quantitative analyses (*n* = 3; **P < 0.01, ****P < 0.0001)

Methyl-beta-cyclodextrin (MβCD) is a widely used reagent for depleting the cholesterol in the plasma membrane of cells. Here, the effects of MβCD at different concentrations on the number of erythrocytes and the cholesterol level in the plasma membrane of erythrocytes were evaluated. Clearly, MβCD could cause the degradation of erythrocytes in a concentration-dependent manner and reach a statistically significant decrease at a concentration of larger than 1.6 mM (Fig. 7A). Flow cytometric quantification found that MβCD could induce a concentration-dependent (statistically significant at 0.8 and 1.6 mM) decrease in cholesterol of the plasma membranes of both normal (Fig. 7B) and sickle (Fig. 7C) erythrocytes as well as in cholesterol of membrane ghosts of both normal and sickle erythrocytes (Fig. 7D), confirming the cholesterol-depleting effect of MβCD.

**Fig. 8:**
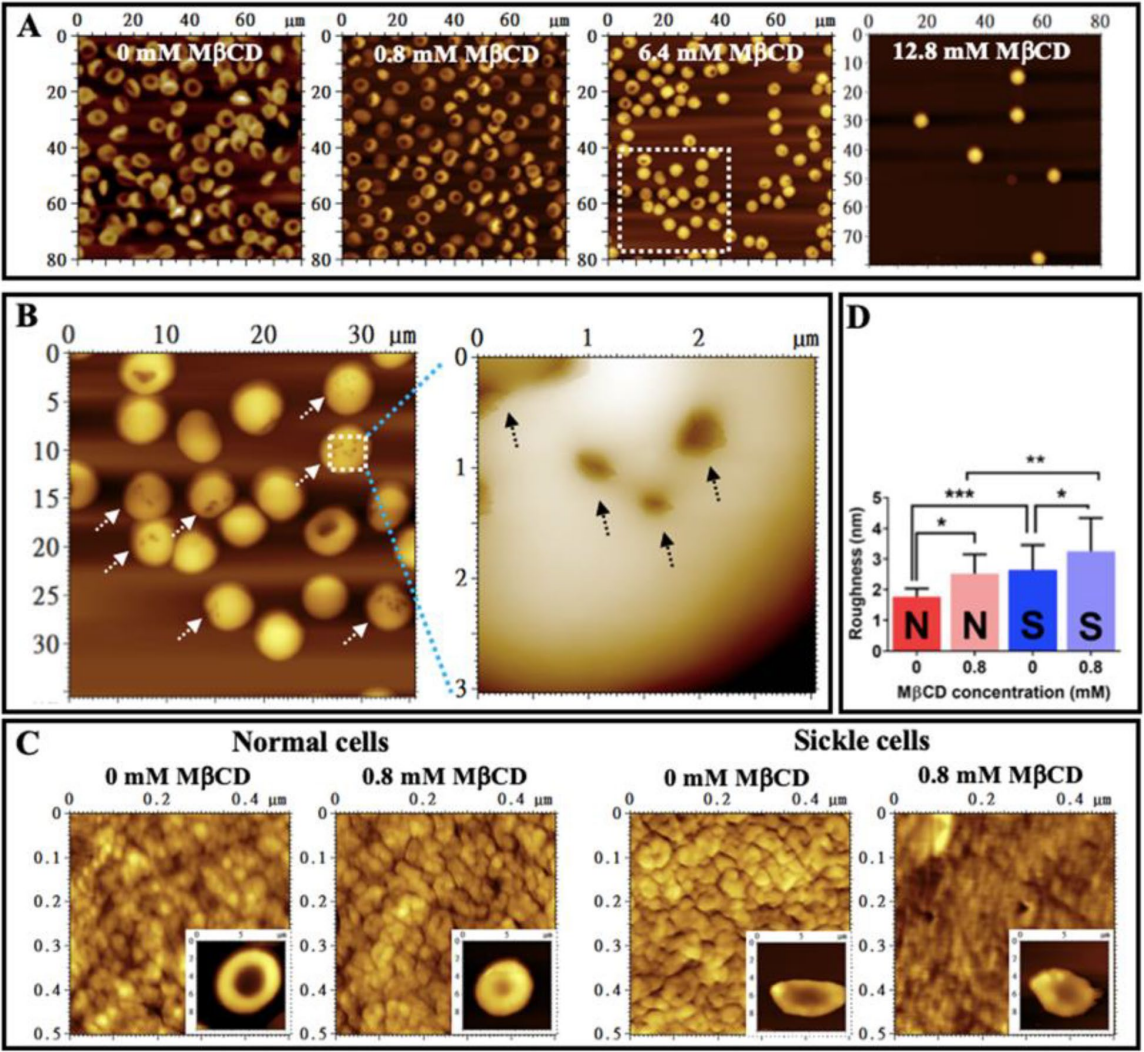
Effects of MβCD on erythrocytes detected by AFM after glutaraldehyde fixation. **A** AFM topographical images (80 μm × 80 μm) of normal erythrocytes treated with MβCD at 0, 0.8, 6.4, and 12.8 mM, respectively for 1h at 37 °C (from left to right). **B** AFM topographical images of entire erythrocytes (40 μm × 40 μm) treated with 6.4 mM MβCD and an enlarged area from the dashed square (3 μm × 3 μm). The arrows indicate the large pits in the surface of erythrocytes. **C** AFM topographical images of local surfaces (0.5 μm × 0.5 μm) of normal and sickle erythrocytes treated with or without 0.8 mM MβCD for 1h at 37 °C, respectively. Insets display the entire cells (10 μm × 10 μm). **D** Quantitative analysis of the surface roughness of normal and sickle erythrocytes treated with or without MβCD (*n* = 25; *P < 0.05, **P < 0.01, ***P < 0.001). The marks N and S represent the normal and sickle cell samples, respectively

The following phenomena are observed in AFM topographical images of entire erythrocytes treated with MβCD: (a) With the increasing of MβCD concentration (from 0 mM to 12.8 mM) the number of erythrocytes decreased gradually (Fig. 8A); (b) more and more biconcave erythrocyte transformed into spherical erythrocytes (Fig. 8A) whereas most erythrocytes treated with 0.8 mM MβCD still remained in a biconcave shape (panel 2 of Fig. 8A); (c) Large pits (indicated by arrows in Fig. 8B) appeared in the surfaces of many erythrocytes treated by MβCD at a relatively high concentration (e.g., 6.4 mM); (d) 0.8 mM MβCD treatment did induce significant changes in shape of erythrocytes (the length to width ratio: 1.019 ± 0.124 vs. 1.004 ± 0.131 for untreated and MβCD-treated normal cells, respectively and 1.236 ± 0.287 vs. 1.207 ± 0.220 for untreated and MβCD-treated sickle erythrocytes, respectively; *n* = 50, *P* > 0.05); (e) 0.8 mM MβCD treatment also caused the increase in cell-surface roughness for both normal and sickle erythrocytes as showed by AFM images of local surfaces of erythrocytes (Fig. 8C) and quantitative analysis (Fig. 8D; the average cell-surface roughness is 1.8 ± 0.3 nm and 2.5 ± 0.6 nm for normal and sickle cells without treatments, respectively, and 2.6 ± 0.8 nm and 3.3 ± 1.1 nm for normal and sickle cells treated with 0.8 mM MβCD, respectively). MβCD-induced cholesterol depletion was responsible for the cell-surface roughening and membrane damage (e.g., the large pits) which further contributed to the shape change and cell degradation probably due to the increase of membrane permeability. Since 0.8 mM MβCD could induce a statistically significant cholesterol depletion but no statistically significant cell degradation and no obvious changes in the entire morphology/shape of erythrocyte, this concentration (0.8 mM) of MβCD was used in the following AFM experiments.

### Cholesterol depletion by MβCD causes an increase in roughness and a decrease in thickness of membrane ghosts for both normal and sickle erythrocytes

**Fig. 9:**
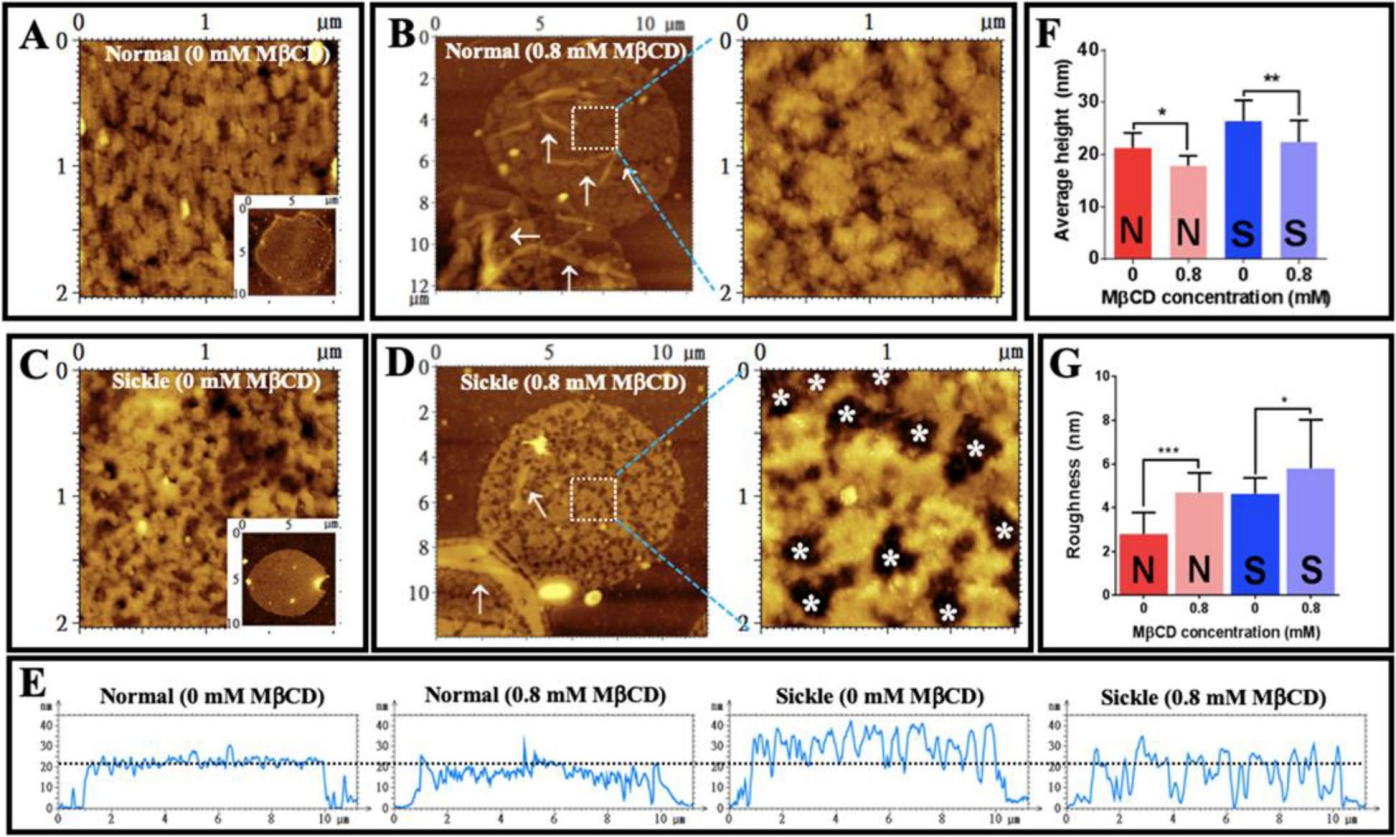
Effect of MβCD on membrane ghosts of normal and sickle erythrocytes (fixed by glutaraldehyde after MβCD treatment). **A, B** Membrane ghosts from normal erythrocytes. **C**, **D** Membrane ghosts from sickle erythrocytes. (**A**) AFM topographical image of a local surface (2 μm × 2 μm) enlarged from an untreated ghost (inset). **B** AFM topographical images of the MβCD-treated ghosts (left, ~ 12 μm × 12 μm) and a local surface (right, 2 μm × 2 μm). **C** AFM topographical image of a local surface (2 μm × 2 μm) enlarged from an untreated ghost (inset). **D** AFM topographical images of the MβCD-treated ghosts (left, ~ 12 μm × 12 μm) and a local surface (right, 2 μm × 2 μm). **E** Height profiles of the cross sections across entire ghosts from normal or sickle cells with or without treatment. (MβCD). **F** Quantitative analysis of average height of ghosts in two layers (*n* = 50). **G** Quantitative analysis of surface roughness (Sa) of ghosts (*n* = 25). The ghosts were treated with 0.8 mM MβCD for 1 h at 37 °C. The white arrows in **B** and **D** indicate the creases on ghosts treated by MβCD. The white asterisks in **D** indicate the large pits with a diameter of ~250–500 nm (231.8 ± 20.4 nm; *n* = 20 cells) in ghosts treated by MβCD. Statistical significance is indicated as follows:*P < 0.05, **P < 0.01, ***P < 0.001. The marks N and S represent the normal and sickle cell samples, respectively in **F** and **G**

After isolation from erythrocytes, membrane ghosts were also treated with 0.8 mM MβCD and imaged by AFM (Fig. 9). Compared with the relatively smooth surfaces of membrane ghosts from normal erythrocytes (Fig. 9A), the surfaces with many small bumps could be observed on membrane ghosts of sickle erythrocytes (Fig. 9C) as also showed in Fig. 5B. After 0.8 mM MβCD treatment, many creases could be observed on membrane ghosts from both normal and sickle erythrocytes (as indicated by the arrows in left panels of Fig. 9B, D) implying the local detachment of membrane ghosts from the underlying cytoskeleton. Compared with the membrane ghosts without MβCD treatment (Fig. 9A, C), the membrane ghosts treated with MβCD (right panels of Fig. 9B, D) had a larger roughness which was confirmed by the quantitative analysis (Fig. 9G; the average roughness is 2.8 ± 1.0 nm and 4.6 ± 0.7 nm for normal and sickle cells without treatments, respectively and 4.7 ± 0.9 nm and 5.8 ± 2.2 nm for normal and sickle cells treated with 0.8 mM MβCD, respectively). Moreover, after MβCD treatment, relatively big pits (indicated by the white asterisks) appeared in the membrane ghosts of sickle erythrocytes (right panel in Fig. 9D). On the other hand, MβCD treatment induced a ~3.4-nm reduction in average height of normal cell membrane ghosts (21.3 ± 2.8 nm for non-treatment vs. 17.9 ± 1.9 nm for treatment) and a ~4.0-nm reduction in average height of sickle cell membrane ghosts (26.4 ± 4.0 nm for non-treatment vs. 22.4 ± 4.1 nm for treatment), respectively according to the height profiles of cross sections (Fig. 9E) and the quantitative analysis (Fig. 9F).

### Sickle erythrocytes are stiffer than normal erythrocytes and cholesterol depletion by MβCD induces a larger decrease in stiffness of sickle cells than that of normal erythrocytes detected by AFM

**Fig. 10:**
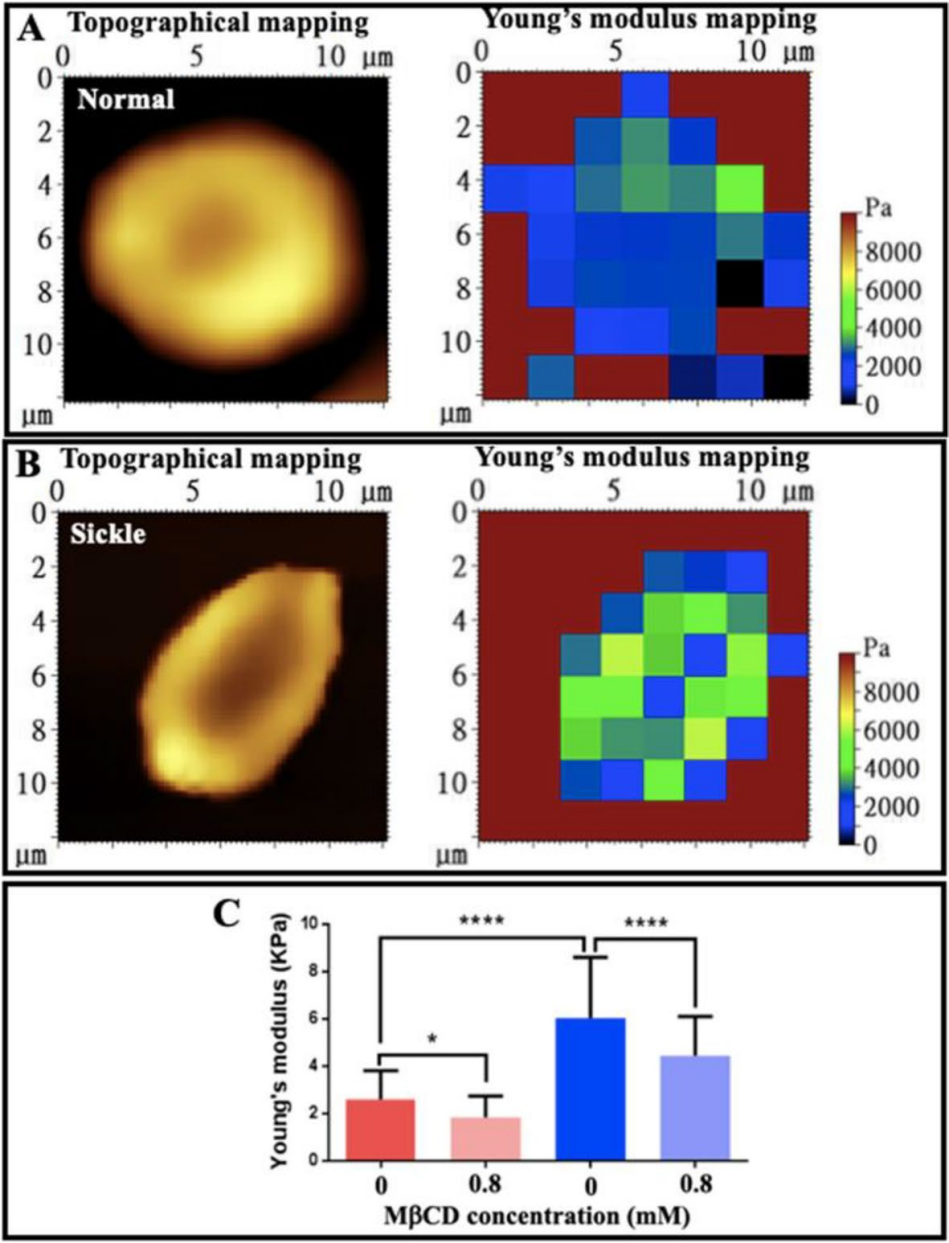
AFM detection of the stiffness of individual erythrocytes in PBS buffer. **A** A representative normal erythrocyte. **B** A representative sickle erythrocyte. Left: topographical mapping; right: Young’s modulus mapping. **C** Quantitative analysis of Young’s modulus (*n* = 100; *P < 0.05, ****P < 0.0001). All erythrocytes in this experiment were not fixed by a fixative

Finally, AFM was recruited to detect/measure the rigidity/stiffness of both normal and sickle erythrocytes treated with or without MβCD. Due to the potential influence of a fixative on cell stiffness, all erythrocytes for stiffness measurement were not fixed by glutaraldehyde in this experiment. By using the force measurement function (force spectroscopy) of AFM, both the topographical mapping and Young’s modulus mapping were performed on the same single erythrocytes from healthy subjects and sickle cell anemia patients (Fig. 10). Compared with the normal erythrocytes (Fig. 10A), sickle erythrocytes (Fig. 10B) had a much higher average Young’s modulus (6.036 ± 2.577 kPa for sickle cells vs. 2.597 ± 1.221 kPa for normal cells; Fig. 10C) implying that sickle cells are ~2.3-fold stiffer than normal erythrocytes. Upon 0.8 mM MβCD treatment, the average Young’s moduli of both normal and sickle erythrocytes significantly decreased to 1.847 ± 0.909 kPa for normal erythrocytes and 4.456 ± 1.659 kPa for sickle cells, respectively (Fig. 10C).

## Discussion

The significant decreases in plasma lipids (particularly cholesterol, generally named “hypocholesterolemia”) in sickle cell anemia patients have been reported in many studies [16–19]. This phenomenon was confirmed in the current study by detecting lower cholesterol and phospholipid levels in plasma/lipoproteins from sickle cell anemia (SCA) patients compared with healthy subjects (Fig. 1). Interestingly, in SCA patients we also detected the decrease in mass/activity of plasma phospholipid transfer protein (PLTP) (data not shown) which functions as a bridge exchanging phospholipids between different types of plasma lipoproteins (e.g., high-density lipoprotein or HDL and low-density lipoprotein or LDL) and plays critical role in lipid metabolism and cardiovascular diseases (e.g., atherosclerosis) [20, 21]. It implies that the impairment of the PLTP function probably was involved in the reduction of plasma phospholipid levels in SCA patients. The role/involvement of PLTP in the lipid/lipoprotein metabolism during SCA development is worth being further explored in the future.

**Fig. 11:**
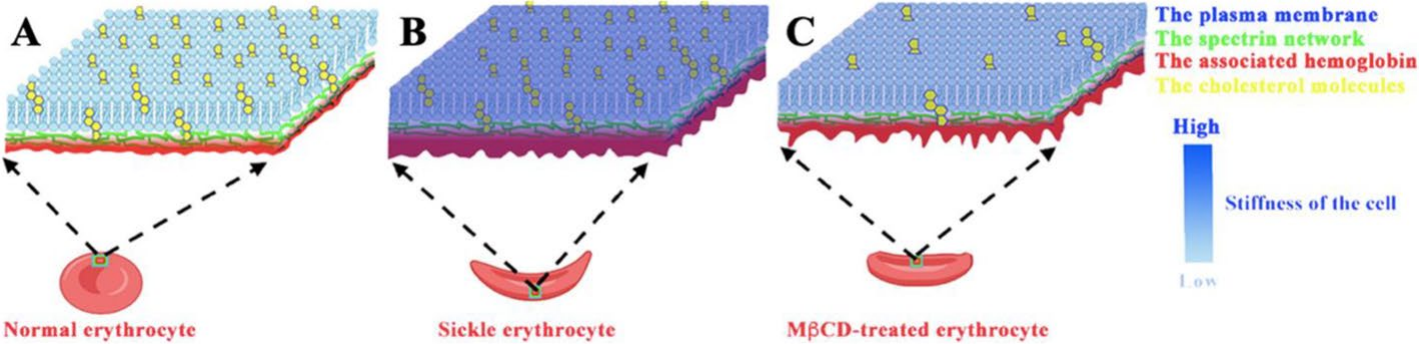
Schematic diagram showing the differences between normal erythrocytes and sickle cells, as well as the effects of MβCD treatment. Compared with normal erythrocytes (**A**), sickle cells had a thicker hemoglobin layer (in red) associated with the plasma membrane (in blue) and the spectrin skeleton/network (in green), rougher outer (not shown here) and inner surfaces of the cell envelope, and a higher stiffness of the entire cell (in a gradient blue; the color scale bar shows the different extents of cell stiffness from high to low) (**B**). After MβCD treatment at a relatively mild concentration, many cholesterol molecules (in yellow) in the plasma membrane were removed/depleted, the thickness of the membrane-associated hemoglobin layer (or the cell envelope) decreased, the roughness of the outer (not shown here) and inner surfaces of the cell envelope increased, and the stiffness of the entire cell also reduced for both normal and sickle erythrocytes (**C**) while the MβCD-induced changes on sickle cells were stronger than the changes on normal cells (not shown here)

By using atomic force microscopy (AFM), the following findings about sickle cells are achieved (Fig. 11): (1) the surface of sickle cells is rougher than that of normal erythrocytes (Fig. 2); (2) the outer and inner surfaces of sickle cell membrane ghosts are rougher than those of normal erythrocytes (Figs. 5D and 6D and E); (3) the sickle cell ghost is thicker than the normal erythrocyte ghost both in two layers (26.4 ± 4.0 nm and 21.3 ± 2.8 nm for sickle and normal ghosts, respectively; Figs. 4F and 5C) and in one layer (13.9 ± 1.6 nm and 11.3 ± 1.2 nm for sickle and normal ghosts, respectively; Fig. 6C); (4) sickle cells are stiffer than normal erythrocytes (Fig. 10; 6.036 ± 2.577 kPa for sickle cells vs. 2.597 ± 1.221 kPa for normal cells which is at the same order, e.g., 0.1–10 kPa, for most types of animal cells [22, 23]).

The membrane-associated hemoglobin S (HbS) proteins/aggregates were responsible for the higher thickness (an approximately 23–24% or ~2.5 nm increase in thickness of the cell envelope) of sickle cell ghosts compared with normal ghosts (i.e., the abovementioned finding 3). Our SDS-PAGE data also shows that the isolated membrane ghosts from sickle cells contained more hemoglobin proteins than those from normal erythrocytes (Fig. 3). Actually, the hemoglobin binding to normal erythrocyte membrane [24–26] and the enhanced hemoglobin-membrane association/interaction in sickle erythrocytes [26–29] have long been detected by different techniques except AFM. It has been reported that hemoglobin potentially binds to erythrocyte membrane or the underlying spectrin network via interacting with the cytoplasmic domain of band 3 protein [30]. The membrane-associated hemoglobin proteins/aggregates might also contribute to the greater roughness of the outer and inner surfaces of sickle cell membranes (i.e., the abovementioned findings 1 and 2). The rigid aggregates (possibly tiny fibers) of membrane-bound hemoglobin S (HbS) proteins might distort the plasma membrane of sickle cells or the isolated sickle cell ghosts, resulting in the greater surface roughness or the formation of many small bumps (Fig. 5B). It is probably like a layer of gravels (rigid hemoglobin aggregates) under a carpet (the plasma membrane or membrane ghost) causing small bumps in the outer and inner surfaces of the carpet.

Previous AFM studies have revealed that sickle cells are stiffer than normal erythrocytes [7, 8], coinciding with our finding (i.e., the abovementioned finding 4). The cytosolic hemoglobin (HbS) aggregates/fibers have been regarded as the contributor for the rigidity of sickle cells [4, 5]. Based on the results from micropipette aspiration tests on individual erythrocytes, however, Evans & Mohandas suggested that membrane-associated hemoglobin is a major determinant of sickle erythrocyte rigidity [31]. Via simple optical observations, we found that after the removal of cytosolic content (hemoglobin S aggregates/fibers), most sickle-shaped erythrocytes finally became the wafer-shaped membrane ghosts (Fig. 4C, D). This phenomenon indicates that it is the hemoglobin S aggregate/fiber instead of the cell envelope (e.g., the membrane skeleton) that maintains the sickle-like shape of sickle cells, implying the strong rigidity of the hemoglobin S aggregate/fiber and its major contribution to the stiffness of a whole sickle erythrocyte. Interestingly, however, the transformation of sickle-shaped cells into wafer-shaped membrane ghosts relatively lagged behind in comparison with the transformation of normal erythrocytes (Fig. 4A). Moreover, some sickle cells temporarily remained in a sickle-like shape after the removal of hemoglobin S aggregates/fibers (Fig. 4B). The two phenomena imply that the cell envelope may also contribute to the shape maintenance and the stiffness of sickle cells. Therefore, we hypothesized that both the cytosolic hemoglobin fibers and the cell envelope (including the plasma membrane, the membrane skeleton or spectrin network, and the membrane-associated hemoglobin) contribute to the shape maintenance and the biomechanical properties (e.g., rigidity/stiffness) of sickle erythrocytes.

To further elucidate the involvement of the cell envelope in the stiffness of sickle cells, methyl-β-cyclodextrin (MβCD), a putative cholesterol-depleting reagent which was reportedly able to change the biomechanical properties of the plasma membrane and the membrane association of the membrane skeleton, was subsequently recruited. The following findings are achieved (Fig. 11): (a) MβCD can deplete cholesterol in the plasma membrane of erythrocytes at relatively low concentration (≤ 0.8 mM) and destroy the cell envelope (e.g., the formation of large pits) or even degrade entire erythrocytes at a high concentration (≥ 1.6 mM) for both normal and sickle erythrocytes (Figs. 7 and 8A, B); (b) the surface of normal/sickle erythrocytes treated by MβCD is rougher than that of untreated cells (Fig. 8C, D); (c) the MβCD-treated normal/sickle cell ghosts are rougher and thinner than untreated ghosts (Fig. 9; 26.4 ± 4.0 nm before MβCD treatment vs. 22.4 ± 4.1 nm after treatment for two layers of sickle ghosts and 21.3 ± 2.8 nm before treatment vs. 17.9 ± 1.9 nm after treatment for two layers of normal ghosts, respectively); (d) the MβCD-treated normal/sickle erythrocytes are softer than untreated cells, and MβCD can induce a more significant decrease in stiffness of sickle cells than that of normal erythrocytes (Fig. 10; 6.036 ± 2.577 kPa before MβCD treatment vs. 4.456 ± 1.659 kPa after treatment for sickle cells and 2.597 ± 1.221 kPa before treatment vs. 1.847 ± 0.909 kPa after treatment for normal cells, respectively).

It is well known that cholesterol is the major component of lipid raft, a cholesterol-rich and sphingolipid-rich membrane microdomain in the plasma membrane of animal cells (including erythrocytes [32, 33]) functioning as a platform for signal transduction and as a linkage between the cytoskeleton and the plasma membrane [34–37]. In erythrocytes, the association of spectrin, actin and other spectrin-interacting proteins (e.g., protein 4.2) with lipid raft in the plasma membrane has been reported [38]. It means that cholesterol depletion by MβCD is able to trigger the disassociation of the membrane skeleton (spectrin meshwork and spectrin-interacting proteins in erythrocytes) from the plasma membrane by disrupting lipid rafts [39, 40] (actually, MβCD is widely used as a lipid raft-disrupting reagent in the lipid raft research field). In this study, the MβCD-induced cholesterol depletion caused the disassociation of a part of the membrane skeleton and the membrane/skeleton-associated hemoglobin, therefore resulting in the decreases in thickness of normal/sickle cell ghosts and in stiffness of normal/sickle cells.

It seems that MβCD exerted a similar effect on both normal and sickle erythrocytes due to the nonspecific depletion of cholesterol from cells. The ~15% decrease in thickness of the cell envelope and the ~25–30% decrease in stiffness of normal/sickle erythrocytes induced by MβCD imply that the cell envelope was involved in the stiffness of normal/sickle erythrocytes. Interestingly, however, MβCD caused the changes of sickle cells to a greater extent compared with normal cells (~2.0 nm and ~1.7 nm decreases in thickness of the cell envelope for sickle and normal cell ghosts, respectively in Fig. 9F; ~1.58 kPa and ~0.75 kPa decreases in cell stiffness for sickle and normal cells, respectively in Fig. 10C), implying that the cell envelope (particularly the membrane/cytoskeleton-associated hemoglobin) contributes to the stiffness of sickle cells to a greater extent than normal cells.

## Conclusions

In this study, we aimed to evaluate the relationship between the cell envelope and the biomechanical properties of sickle cells from sickle cell anemia (SCA) patients mainly by using atomic force microscopy to detect the entire erythrocytes or their membrane ghosts. We found:

(i) Lower lipid (particularly cholesterol) levels and apoB levels in sera/lipoproteins of SCA patients than the healthy controls, confirming previous reports;
(ii) Rougher inner surface than the outer surface for the cell envelopes of both normal and sickle erythrocytes whereas rougher surface (for both inner and outer surfaces) of sickle erythrocytes/ghosts than normal erythrocytes/ghosts, implying the potential existence of a membrane-associated hemoglobin layer in the inner surface of the cell envelope;
(iii) Higher hemoglobin content and thicker height of sickle erythrocyte membrane ghosts than those of normal ghosts (an average thickness of the membrane ghost monolayer: 13.9 ± 1.6 nm and 11.3 ± 1.2 nm for sickle and normal erythrocytes, respectively), implying the greater thickness of the cell envelope (or membrane-associated hemoglobin layer in the cell envelope) of sickle erythrocytes than normal cells;
(iv) An increase in roughness and a decrease in thickness of membrane ghosts for both normal and sickle erythrocytes (also the appearance of many big pits in sickle cell membrane ghosts) after cholesterol depletion by 0.8 mM MβCD (a widely used cholesterol-depleting reagent for cellular experiments), implying that the cholesterol depletion by MβCD could significantly influence the structure of the cell envelope by modifying the plasma membrane and detaching (at least partially) the membrane-associated hemoglobin or skeleton from the cell envelope; and
(v) Much higher stiffness of sickle erythrocytes than that of normal cells (an average Young’s modulus: 6.036 ± 2.577 kPa and 2.597 ± 1.221 kPa for sickle and normal erythrocytes, respectively) which could be significantly lowered by MβCD treatment, implying that MβCD-induced changes in the cell envelope or in the membrane-associated hemoglobin layer (consisting of rigid aggregates/fibers of membrane-bound hemoglobin proteins) may influence cellular stiffness.

Taken together, the findings support that besides the intracellular polymerization of hemoglobin, the cell envelope containing the membrane-associated hemoglobin also is responsible for the biomechanical properties (e.g., stiffness and shape maintenance) of sickle erythrocytes. This study may provide important information for understanding the mechanisms of the elevated rigidity/stiffness of sickle erythrocytes.

## Methods

### Sample preparations

Human red blood cells (RBCs) or erythrocytes were prepared from blood drawn from 4 sickle cell anemia patients and 3 healthy human subjects. The blood was collected in a tube containing potassium EDTA and centrifugated at 2000 rpm for 5 min to separate erythrocytes (the cell pellets) and sera (the supernatants). The serum samples were used immediately for the lipid test while the erythrocyte samples were stored at 4 °C at most for one week. Before experiments, intact erythrocytes were washed twice with PBS buffer and resuspended in PBS. Ethics approval for the study was obtained from the Research Ethics Board of Interfaith Medical Center in Brooklyn, the informed consent was obtained for each participant, and the blood sample collection from live subjects was performed in full compliance with the National Institute of Health (NIH) Guide for human/animal Care and Use of Laboratory Animals and Downstate Medical Center guidelines on human subjects (Human IRB Protocol number: 08-154).

For preparation of erythrocyte membrane ghosts, the erythrocytes in PBS were centrifugated at 3000 rpm for 5 min at 4 °C, resuspended in double distilled water and shaken for seconds, and spun at 10,000 rpm at 4 °C for 10 min. The pellets were resuspended in PBS buffer and spun at 10,000 rpm at 4 °C for 10 min; this process was repeated until the red color of the solution disappeared; finally, the pellets were collected, resuspended in PBS, and subjected to the following experiments.

### Lipid profiling via fluorescence spectrometry

The concentrations of total cholesterol (TC), phospholipids (PL), and triglyceride (TG) in serum were measured by enzymatic colorimetric methods using corresponding reagents (Wako Diagnostics) as previously reported [41]. Briefly, a serum of 3 μL for TC/PL or 5 μL for TG was mixed with 100 μL reagent in a well of the 96-well transparent plate, reacted at 37 °C for 10–15 min, and detected by a SpectraMax 250 plate Reader (Molecular Devices) at 600 nm for TC and PL or at 495 nm for TG. Standards (200 mg/dL for TC/TG or 300 mg/dL for PL) were used for the determination of the concentrations.

### Lipoprotein distribution assay via FPLC and fluorescence spectrometry

The major lipoproteins (the apoA-containing HDL and the apoB-containing non-HDL) was separated by a fast protein liquid chromatography (FPLC) system (Pharmacia Biotech) as previously reported [41, 42]. Briefly, a 350-μL aliquot of pooled serum from 3 healthy controls or 4 patients was loaded onto an FPLC column (Superose 10/300 GL) and eluted with FPLC system buffer (50 mM tris buffer, pH 7.4) at a constant flow rate of 0.35 mL/min. Approximately 36 fractions were collected in tubes. The lipid levels in each fraction were determined by the similar method to lipid profiling (here, ~80 μL of each fraction was incubated with 100 μL reagent).

### Determination of apoA-I and apoB mass in serum via western blotting

The levels of apoA-I and apoB in serum were determined by western blotting. Serum was diluted by mixing 5 μL serum with 45 μL PBS buffer. The samples were prepared by mixing 5 μL diluted serum with 10 μL loading buffer and 35 μL PBS. Approximately 10 μL samples pre-inactivated by heat was separated by SDS-PAGE first at 40 V and then at 100 V on precast 4-20% polyacrylamide gels (Bio-Rad Laboratories Inc.), transferred at 100 V for 1 h onto 0.45 μm nitrocellulose membrane (Bio-Rad Laboratories Inc.) on ice, blocked with 5% nonfat milk in 1× TBS-T buffer (5 N NaCl, 0.5 M EDTA at pH 8, 1 M Tris HCl at pH 7.5, and 0.8% Tween-20 in double distilled water for 10× TBS-T buffer) in a cold room overnight, immunoblotted at room temperature successively with 5% BSA-containing primary antibodies (mouse anti-human apoA-I mAb and mouse anti-human apoB mAb) and 5% nonfat milk-containing secondary antibodies (goat anti-rabbit IgG-HRP for apoA/apoB and rabbit anti-mouse IgG pAb-HRP for PLTP; Novus Biologicals). Next, the film was incubated with SuperSignal West Pico Chemiluminescent Substrate (Thermo Scientific, Rockford, IL) in a dark room and immediately exposed. After each of the abovementioned steps between milk blockage and chemiluminescence, the film was washed at least 3 times with 1× TBS-T buffer.

### Fluorescence staining and detection via flow cytometry

The erythrocytes with or without methyl-β-cyclodextrin (MβCD) treatment were fixed with 0.5% glutaraldehyde at room temperature for 30 min, washed with PBS for three times, stained with 50 μg/mL filipin (specific fluorescent dye for cholesterol) for 30 min at 37 °C, washed again with PBS for three times, and then subjected to flow cytometry (FACSCalibur, BD Biosciences, USA).

### Atomic force microscopy (AFM)

Prior to AFM detection, the erythrocytes or membrane ghosts were observed by an LSM710 confocal microscope (Carl Zeiss, Oberkochen, Germany) equipped with an inverted microscope and a Zeiss 40× (0.75 NA) Plan-Neofluar objective lens, and only the differential interference contrast (DIC) images were obtained/displayed.

For AFM imaging, intact erythrocytes and membrane ghosts with or without MβCD treatment were incubated in PBS for 60 min at room temperature in petri dishes precoated with poly-L-lysine. After fixing with 0.5% glutaraldehyde at room temperature for 30 min and gently washing with PBS for three times, the petri dishes with erythrocytes or membrane ghosts were mounted on the sample stage of AFM for imaging. In order to observe the inner surface of the plasma membrane of erythrocytes, a lysis-squirting method was applied to expose the inner leaflet of membrane ghosts [43, 44]. Briefly, the erythrocytes were attached onto the poly-L-lysine-coated substrate in a petri dish and were sprayed at a rate of 0.8 mL/s at an angle of 25 °C with a 25G needle. The resulting shear force was utilized to shear off the upper membrane exposing the cytoplasmic surface of the bottom membrane. After washing with distilled water three times, fixing with 0.5% glutaraldehyde at room temperature for 30 min, and washing again for three times, the samples were mounted on the sample stage of AFM for imaging.

An Agilent 5500 AFM (Agilent, USA) equipped with a scanner of 90 μm × 90 μm × 10 μm was utilized. For topographical detection, the samples prefixed with glutaraldehyde were imaged by tapping mode AFM in air. The SiO_2_ probes (qp-BioAC, Nanosensors, USA) with an end radius of smaller than 10 nm and a spring constant of 0.06 N/m, which are made of a quartz-like material, were used for imaging erythrocytes and membrane ghosts. The AFM probe was scanned across the sample surface at 0.5–1 Hz with a tracking force of 50–100 pN. The instrument-equipped software (Picoimage 6.2) was utilized to analyze AFM data. The roughness and thickness of erythrocytes and membrane ghosts in topographical images were calculated after being flattened by one level. The following equation was utilized to calculate the average surface roughness deviations (Sa) of erythrocytes or membrane ghosts in AFM topographical images (size: 0.5 μm × 0.5 μm or 1 μm × 1 μm).

$$\frac{\textbf{1}}{n_x{\boldsymbol{n}}_{\boldsymbol{y}}}\sum_{\textbf{j}=\textbf{1}}^{{\textbf{n}}_{\textbf{x}}}\ \sum_{\textbf{i}=\textbf{1}}^{{\textbf{n}}_{\textbf{y}}}\ \left|\boldsymbol{\upeta} \left({\textbf{x}}_{\textbf{i}},{\textbf{y}}_{\textbf{j}}\right)\right|$$

where n_x_/n_y_ and i/j represent the total number of data points and the indices in x/y direction, respectively; *η*(x_i_, y_j_) is the surface heights representing the height of each point based on the mean plane.

For the detection of Young’s modulus, the cells without glutaraldehyde fixation were measured by contact mode AFM in PBS. For force curve mapping, the probe tips approached cell surfaces at 200 nm/s, pressed sample surfaces with a loading force of 1 nN, and retracted from the surface at 200 nm/s. The cantilever spring constant (~0.06 N/m) was measured by using a thermal K method program, and the deflection sensitivity of cantilever was calibrated on a mica in PBS prior to the force curve mapping. After the force curve mapping, the AFM probe was scanned across the sample surface at ~0.2 Hz with a tracking force of 50–100 pN to obtain the topographical mapping of the same samples. The Young’s modulus was calculated from force-indentation curve (an indentation depth of 8% of the sample height was applied) which was extracted from the force-vs-distance curves via the Hertz model by using the instrument-equipped PicoView 1.14 software as previously reported [23, 45].

### Statistical analysis

All data from at least three independent experiments were expressed as mean ± SD. Statistical analysis was performed using Student’s *t*-test between two groups or one-way ANOVA among multiple groups to determine the significant difference (*P*<0.05 was considered statistically significant).

## Supplementary Information

**Additional file 1.** Full length blots.

## Data Availability

All data generated or analyzed during this study are included in this study. Full length blots can be found in Additional File 1.

## Abbreviations

AFM: Atomic force microscopy
ApoA-1: Apolipoprotein A-1
ApoB: Apolipoprotein B
DIC: Differential interference contrast
HbA: Hemoglobin A
HbS: Hemoglobin S
HDL: High-density lipoprotein
FPLC: Fast protein liquid chromatography
LDL: Low-density lipoprotein
MβCD: Methyl-β-cyclodextrin
PBS: Phosphate-buffered saline
PL: Phospholipids
PLTP: Phospholipid transfer protein
RBCs: Red blood cells
SCA: Sickle cell anemia
SDS-PAGE: Sodium dodecyl sulfate - polyacrylamide gel electrophoresis
TC: Total cholesterol
TG: Triglyceride
VLDL: Very low-density lipoprotein
