# Supplementary material for: Atomic force microscopy reveals involvement of the cell envelope in biomechanical properties of sickle erythrocytes
Source: BMC Biol. 2023 Feb 13;21:31. doi: 10.1186/s12915-023-01523-3 (PMC9926656; doi:10.1186/s12915-023-01523-3)

The original, uncropped gels/blots

1. **The original, uncropped blots for ApoA-1 (MW = ~28.3 kDa) in Fig. 1C (the dashed red box indicates the cropped blots used in the paper).**

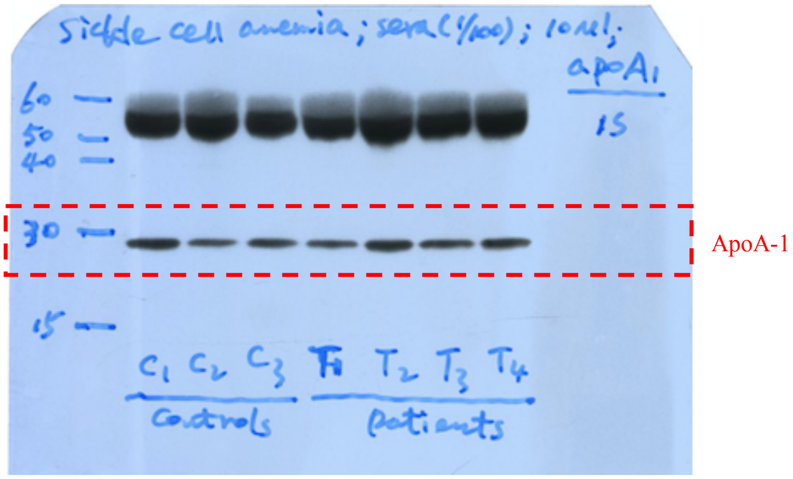

2. **The original, uncropped blots for ApoB (the MW of ApoB-100 is ~510 kDa) in Fig. 1C (the dashed red box indicates the cropped blots used in the paper).**

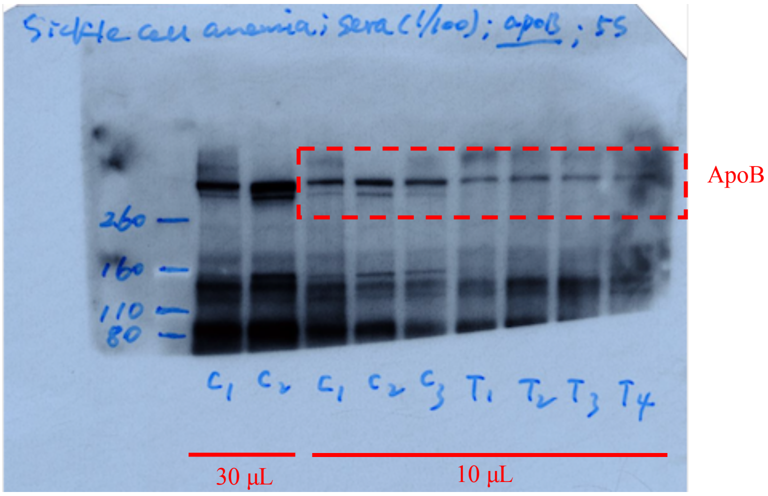

Supplement: Supplementary file 1 — Additional file 1. Full length blots. [file 12915_2023_1523_MOESM1_ESM.pdf]
